# Antibiotic Resistance and Virulence Profiles of Gram-Negative Bacteria Isolated from Loggerhead Sea Turtles (*Caretta caretta*) of the Island of Maio, Cape Verde

**DOI:** 10.3390/antibiotics10070771

**Published:** 2021-06-24

**Authors:** Matilde Fernandes, Miguel L. Grilo, Carla Carneiro, Eva Cunha, Luís Tavares, Juan Patino-Martinez, Manuela Oliveira

**Affiliations:** 1 CIISA-Centro de Investigação Interdisciplinar em Sanidade Animal, Faculdade de Medicina Veterinária, Universidade de Lisboa, Av. da Universidade Técnica, 1300-477 Lisboa, Portugal; cfernandes.matilde@gmail.com (M.F.); miguelgrilo@fmv.ulisboa.pt (M.L.G.); carlacarneiro@fmv.ulisboa.pt (C.C.); evacunha@fmv.ulisboa.pt (E.C.); ltavares@fmv.ulisboa.pt (L.T.); 2 Veterinários Sem Fronteiras, Av. Da Universidade Técnica, 1300-477 Lisboa, Portugal; 3 Maio Biodiversity Foundation (FMB), Cidade Porto Inglês, Ilha do Maio 6110, Cape Verde; juan.patino@fmb-maio.org

**Keywords:** *Caretta caretta*, island of Maio, antimicrobial resistance, bacterial virulence factors, One Health

## Abstract

Previous studies revealed high levels of antimicrobial resistance (AMR) in loggerhead sea turtles (*Caretta caretta*), describing this species as prime reservoir of antimicrobial-resistant bacteria. This study aimed to characterise, for the first time, the AMR and virulence profiles of Gram-negative bacteria isolated from 33 nesting loggerhead turtles of the island of Maio, Cape Verde. Cloacal, oral, and egg content swab samples (*n* = 99) were collected and analysed using conventional bacteriological techniques. *Shewanella putrefaciens*, *Morganella morganii,* and *Vibrio alginolyticus* were isolated from the samples under study. The isolates obtained from this loggerhead subpopulation (North-East Atlantic) revealed lower levels of AMR, compared with the results of studies performed in other subpopulations (e.g., Mediterranean). However, the detection of resistance to carbapenems and multiple antimicrobial resistance indices higher than 0.20, raises concern about the potential association of these animals to points of high antimicrobial exposure. Furthermore, virulence phenotypic characterisation revealed that the isolates presented complex virulence profiles, including the ability to produce biofilms. Finally, due to their pathogenic potential, and considering the evidence of illegal consumption of turtle-related products on the island of Maio, the identified bacteria may represent a significant threat to public health.

## 1. Introduction

According to the World Health Organization (WHO), antimicrobial resistance (AMR) is one of the most critical threats to human health, food safety, and communities’ development worldwide [1].

Loggerhead sea turtles (*Caretta caretta*) have been proposed as prime reservoirs for antimicrobial-resistant bacteria (ARB) due to the species’ unique ecological and physiological characteristics, including a diverse omnivorous diet, long lifespan, and high site fidelity to coastal nesting habitats [2].

ARB have been mainly studied in live-stranded loggerhead sea turtles from the Mediterranean subpopulation. High levels of resistance were described in *Citrobacter* spp., *Pseudomonas aeruginosa*, *Morganella morganii*, and *Proteus vulgaris*, namely to penicillins and tetracyclines [2,3]. The evidence of resistant strains in nesting sea turtles is of great concern in the context of One Health, due to the potential transmission of these strains to humans, mainly local populations [4,5]. Furthermore, ARB, including *Vibrio* spp., *Morganella* spp., *Aeromonas hydrophila*, *Pseudomonas aeruginosa*, and *Escherichia coli*, isolated from loggerheads, were previously associated with several sea turtles’ diseases [6,7,8].

*Caretta caretta* is globally categorised by the International Union for the Conservation of Nature (IUCN) Red List of Threatened Species as vulnerable [9]. However, the *Caretta caretta* subpopulation of Cape Verde (North-East Atlantic subpopulation) is classified as endangered [10]. Moreover, recent data show that this loggerhead subpopulation might be the largest worldwide, which supports the importance of the archipelago of Cape Verde for the conservation of the species [11]. Furthermore, after the Island of Boa Vista, the island of Maio is, together with the Island of Sal, the most important nesting site in the archipelago [12,13].

Due to the endangered status of the loggerhead colony of the island of Maio, there is an increasing need to study this subpopulation to reinforce conservation and surveillance strategies. To the best of our knowledge, there are no studies available describing the antimicrobial-resistance profile of loggerhead’s bacteria of the North-East Atlantic subpopulation. Therefore, this study aims to evaluate the antimicrobial resistance and virulence profiles of Gram-negative bacteria isolated from cloacal, oral, and egg content samples of loggerhead turtles of the island of Maio. The present study also aims to evaluate the impact of these bacterial species on sea turtles’ health and conservation, as well as the underlying public health risks resulting from interactions with these animals and the consumption of turtle-derived products.

Our findings revealed a lower level of AMR for this loggerhead subpopulation compared with other subpopulations addressed in previous studies [2,3,14]. However, the detection of multiple antimicrobial resistance indices higher than 0.20, suggests that Maio’s nesting loggerheads may contact with points of high antimicrobial exposure. Finally, the identified isolates revealed the ability to produce several virulence factors, which raises concern about their pathogenic potential.

## 2. Results

Cloacal (oviductal fluid), oral, and egg content swab samples were collected from 33 animals, comprising a total of 99 samples. From the 99 samples under study, it was possible to obtain a total of 49 isolates (49.49%) from 24 samples.

Considering the culture media from which were isolated and the type of sample, 19 Gram-negative bacilli were selected for further characterization, including *Non Enterobacteriaceae* (*n* = 12) and *Enterobacterales* isolates (*n* = 7). The Gram-negative bacilli were selected based on the animal (respective nesting turtle) and on the type of sample from which they were collected (cloacal, oral, or egg content) and considering the media from which they were obtained, their macro and microscopic characterization, oxidase, and catalase reactions, being representative of all the isolates obtained.

The 19 Gram-negative bacilli were identified using the API 20NE and 20E galleries, allowing to obtain the following results: *Shewanella putrefaciens* (*n* = 5), *Vibrio alginolyticus* (*n* = 4), *Morganella morganii* (*n* = 4), *Enterobacter cloacae* (*n* = 2), *Aeromonas hydrophila/caviae* (*n* = 1), *Brevundimonas vesicularis* (*n* = 1), *Burkholderia cepacia* (*n* = 1), and *Citrobacter* spp. (*n* = 1).

### 2.1. Characterisation of Isolates’ Antimicrobial Resistance Profile

A considerable number of isolates under study (*n* = 13) were resistant or intermediately resistant to at least one of the twelve antimicrobial compounds tested. Higher levels of resistance were detected for tetracyclines (*n*º. of isolates =2), and none of the isolates presented resistance or intermediate resistance to aminoglycosides (amikacin, gentamicin, and tobramycin). Some isolates showed intermediate resistance or resistance to imipenem and enrofloxacin (Table 1).

The bacterial species that showed higher multiple antimicrobial resistance (MAR) index values were *A. hydrophila/caviae* (MAR index value = 0.33), an *E. cloacae* isolate (MAR index value = 0.25), and *B. cepacia* (MAR index value = 0.17) (Table 2).

MAR indices higher than the cut-off value (MAR Index = 0.20) were detected for two isolates (Isolates number 1 and 14) (Table 2). According to Magiorakos et al. [15] classification, no multidrug-resistant (MDR) isolates were detected, as none was non-susceptible to at least three antimicrobial agents of different categories.

### 2.2. Characterisation of Isolates’ Virulence Profile

Regarding virulence characterisation, all isolates were able to produce hemolysins (*n* = 19). Most isolates were able to produce DNases (*n* = 17), lipases (*n* = 15), and biofilms (*n* = 14). Protease production was revealed by more than half of isolates (*n* = 10). Lecithinase (*n* = 4) and gelatinase activities (*n* = 3) were less observed among the tested isolates (Table 3).

Higher virulence profile index (V. Index) values were obtained for *A. hydrophila/caviae* (V. Index value = 0.86), *B. vesicularis* (V. Index value = 0.86) and *S. putrefaciens* (V. Index mean value = 0.80) (Table 2). Isolates were categorised as high threat (MAR index ≥ 0.20; V. Index ≥ 0.50) (*n* = 2), moderate threat (MAR index < 0.20; V. Index ≥ 0.50) (*n* = 9), and no threat (MAR index < 0.20; V. Index < 0.50) (*n* = 8). A total of 11 isolates were classified as a threat (high or moderate) for animal/human host or both.

## 3. Discussion

To the best of our knowledge, this study represents the first description of ARB isolated from nesting loggerhead turtles of the North-East Atlantic subpopulation. The present study also represents the first characterisation of the virulence phenotypic profile of sea turtles’ bacteria, underlining the role of loggerhead turtles as carriers of potentially pathogenic bacteria.

Despite the low number of isolates obtained, the results of this study contribute to the highly required body of evidence on antimicrobial resistance, especially concerning marine wildlife and the marine environments.

For the collection of oral and cloacal samples, Amies swabs were used, being described as reliable, effective, non-traumatic techniques for the isolation and posterior identification of aerobic and facultative anaerobic Gram-negative bacteria of loggerhead sea turtles [3,16]. Oliveira et al. [17] showed that similar collection and transport methods permit the isolation and characterisation of Gram-negative bacteria, even when requiring large distances and processing periods. Moreover, the processing of samples from three different anatomic sites (cloaca, oral cavity, and egg content) allowed the isolation of distinct bacterial species, e.g., the isolation of *A. hydrophila/caviae* and *B. cepacia* from a swab sample of the cloacal and oral cavity, respectively.

In the present study, *S. putrefaciens* was the most prevalent species found in *Caretta caretta*, as previously reported by Blasi et al. [18]. Unexpectedly, no *Pseudomonas* spp. isolates were detected, even though *P. aeruginosa* was one of the most prevalent species isolated from sea turtles in previous studies [4,19]. This finding may emphasise the importance of monitoring AMR in distinct bacterial species regarding the loggerhead subpopulation under study.

All identified bacterial species have been previously isolated from both injured and stranded sea turtles, as well as healthy wild animals [8,18,20], except for *B. vesicularis*, which was isolated for the first time from the oviductal fluid of sea turtles in the present study.

*A. hydrophila/caviae*, *B. cepacia*., *V. alginolyticus* and *Citrobacter* spp. isolated in this study were previously associated with diseases of loggerheads and other sea turtle’s species, including ulcerative stomatitis, ulcerative esophagitis, granulomatous hepatitis, granulomatous nephritis, bronchopneumonia, conjunctivitis, and septicaemia [6,8].

*B. cepacia*, *S. putrefaciens* and *V. alginolyticus*, isolated from egg samples in this study, were previously associated with unhatched eggs [21,22,23]. Craven et al. [22] suggested that opportunist pathogens found in adult females could infect sea turtle eggs and cause embryonic mortality. Therefore, the presence of these bacterial species in the egg content of Maio’s loggerheads may represent a potential threat to successful embryonic development and the overall reproductive success of this loggerhead colony.

Despite the small number of isolates, the level of AMR detected for the isolates in this study is considerably lower compared with previous studies [2,3,4,24]. No MDR bacteria were detected, which is in line with a previous study performed in juvenile hawksbill sea turtles (*Eretmochelys imbricata*) and green turtles (*Chelonia mydas*) from potential coincident feeding grounds [4], but discordant with previous studies in other loggerhead and green turtles’ populations [2,3,14,24].

Following previous studies, higher resistance levels were observed for tetracyclines and lower ones for the aminoglycoside class [2,3,14,17,24]. To the best of our knowledge, no resistance to imipenem was previously described for loggerhead sea turtles’ bacteria. Here, *A. hydrophila/caviae*, *B. vesicularis*, *S. putrefaciens,* and *M. morganii* presented intermediate resistance to imipenem, with *M. morganii* also showing intermediate resistance to meropenem. Regardless of the low incidence of resistance to carbapenems, this finding should be further assessed due to the categorisation of these antimicrobial compounds as last resort options for the treatment of serious Gram-negative infections, being of major importance for human medicine [25].

Sea turtles living in ecosystems affected by humans’ activities are at higher risk of being exposed to antimicrobial environmental pressure [14,26]. The island of Maio is mostly characterised by a pristine environment, being less affected by anthropogenic impacts, such as the discharge of wastewater carrying high levels of antimicrobials, associated with aquaculture, intensive farms, and medical facilities [27,28].

The lower anthropogenic influence observed in the island of Maio may explain the lower levels of AMR in this loggerhead subpopulation compared with other subpopulations addressed in previous studies [2,3,14]. However, the detection of isolates with MAR indices equal to or higher than 0.20 from animals not directly exposed to antimicrobial compounds suggests previous contact to points of high antimicrobial exposure [29]. Therefore, although their nesting sites are less exposed to anthropogenic impacts, Maio’s loggerheads may not be fully protected from antimicrobial environmental pressure. Moreover, the highly migratory nature of sea turtles may expose them to a broad range of marine environments, promoting contact with sources of contamination.

Virulence characterisation showed that the isolates from this study could express virulence traits that may contribute to the evasion of the host immune system and host tissue colonisation and damage [30]. Moreover, most isolates showed the ability to produce biofilms. Biofilm synthesis is one of the most important virulence factors in bacteria, playing a prominent role in AMR. In fact, the antimicrobials concentration required to eliminate bacterial biofilms can be up to 1000-fold higher in comparison with their free-swimming, planktonic counterparts, which makes these microbial communities extremely difficult to control [31,32]. The expression of a high number of virulence factors may play an essential role in the pathogenesis of infections [30].

Considering Singh et al. [33] classification, a considerable number of isolates (*n* = 11) in this study were considered a high threat (MAR Index ≥ 0.20; V. Index ≥ 0.50) or moderate threat (MAR Index < 0.20; V. Index ≥ 0.50) to human/animal host or both. These findings suggest that the isolates obtained from the loggerhead turtles under study can pose a threat as potential pathogens, especially those revealing high virulence indices, such as *A. hydrophila/caviae*, *B. vesicularis,* and *S. putrefaciens*.

*A. hydrophila/caviae*, *V. alginolyticus*, *M. morganii*, *S. putrefaciens*, and *Brevundimonas* spp., isolated in this study, were previously associated with infections in humans, including skin and soft tissue infections, ear and wound infections, urinary tract infections, gastroenteritis, neonatal sepsis, and septicaemia [34,35,36,37,38].

Despite the existent conservation efforts, the slaughter of nesting loggerheads for consumption is frequently practised in the island of Maio [11]. The consumption of turtle-related products represents a risk behaviour for public health, which is supported by the detection of pathogenic and antimicrobial-resistant bacteria isolated from loggerhead turtles in this study.

In conclusion, the lower levels of AMR detected for the Cape Verdean loggerhead subpopulation, compared with the levels identified for other subpopulations, represent positive and encouraging results regarding the current context of AMR. However, the presence of potentially pathogenic Gram-negative bacteria expressing several virulence factors may represent a risk to sea turtles’ health and consequently affect the conservation of this endangered species. Finally, due to their pathogenic potential, the identified bacteria may represent a significant threat to public health. This threat arises mainly through the unsafe and illegal consumption of turtle-derived products.

Further studies are encouraged to characterise the identified bacterial species at the molecular level, as well as to assess the genetic determinants imparting AMR and virulence. It would be equally relevant to further study the resistance mechanisms involved in the isolates’ antimicrobial resistance profile, especially concerning resistance to carbapenems.

## 4. Materials and Methods

### 4.1. Area of Study

**Figure 1:**
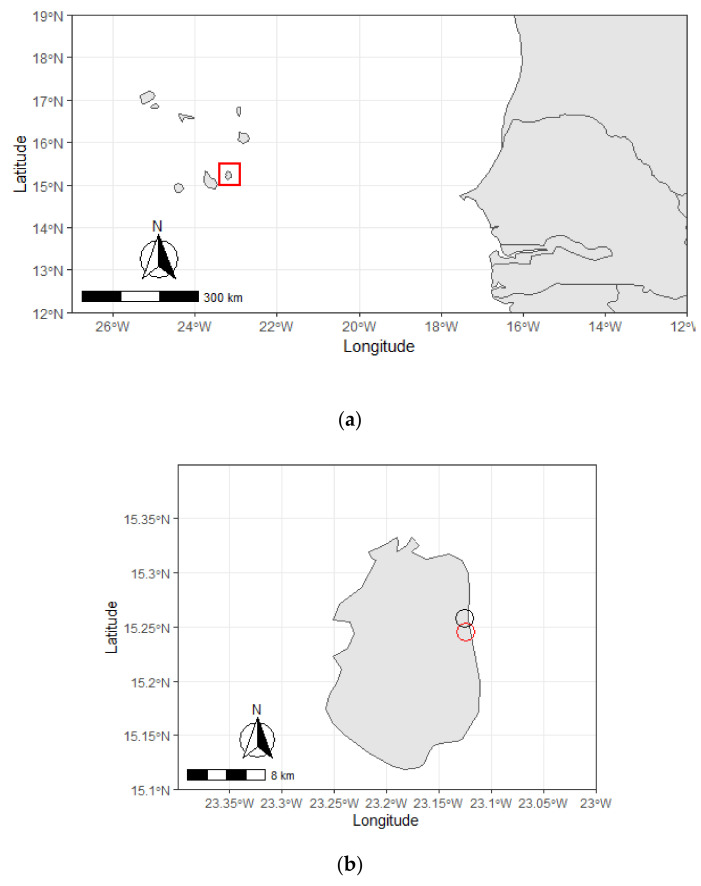
Area of study: **(a)** the archipelago of Cape Verde (15°55′0″ N, 23°55′0″ W), and the island of Maio (highlighted in red). **(b)** The island of Maio (15°13′50″ N 23°09′22″ W) and sampling points: “Praia Gonçalo” (15°15′25.9″ N 23°06′34.5″ W) (black circle) and “Pedro Vaz” (15°14′52.2″ N 23°06′54.5″ W) (red circle). Map created using R package “rnaturalearth” version 0.1.0 [39] and “ggplot2” version 3.3.3 [40] in R-Studio (Version; version 4.0.3).

Samples were collected from loggerhead sea turtles of the island of Maio (15°13′50″ N 23°09′22″ W), the archipelago of Cape Verde (15°55′0′’N, 23°55′0’’W), West Africa (Figure 1a).

The Island of Maio comprehends an area of 269 km^2^ and hosts loggerhead-nesting activity along 38 km of sandy beaches throughout 110 km of coastline [11]. The area of study included the coastal areas of “Pedro Vaz” (15°14′52.2″ N 23°06′54.5″ W) and “Praia Gonçalo” (15°15′25.9″ N 23°06′34.5″ W), namely the beaches “Praiona”, “Cozinha fácil”, and “Areia Preta” (Figure 1b).

### 4.2. Sample Collection

A total of 33 nesting loggerhead sea turtles (*Caretta caretta*) were sampled during August 2019. Oral, cloacal, and egg content samples were obtained from each female turtle, using Amies swabs 1814-002 (VWR, Leuven, Belgium).

The three samples were collected sequentially, in the following order: cloacal, oral, and egg. For cloacal samples, the swab was gently inserted approximately 5 cm into the cloaca, and with a rotational movement, the oviductal fluid from the internal surface was collected.

Oral sampling was performed by opening the rhamphotheca with a previously disinfected (ethylic alcohol 70%) wooden pry bar. The soft tissue of the mouth (tongue and palate) was gently swabbed for approximately 5 s.

For the egg sample, an egg was collected directly from the cloaca without contacting the surrounding environment. A small surface of the shell was sterilised with a fire-heated bistoury, and a circle shape window was cut. A sterilised Pasteur pipette was used to collect approximately 1.5 mL of the egg content (yolk and albumen), which was introduced directly in the transport medium of the Amies swabs. The samples were identified with date, time, type of sample, and flipper tag number and then safely placed in a thermic bag at 4 °C.

After the sampling period, the collected samples were transported to the Microbiology and Immunology Laboratory of the Veterinary Faculty, University of Lisbon, Portugal, for further processing. The following information was collected regarding each sample from each animal: date and time of sampling, local of sample collection, flipper tag identification number, Passive Integrated Transponder (PIT) number (when available), and type of sample (cloaca, oral cavity, and egg content) (See Supplementary Table S1). The handling time did not exceed 5 min, before and after which the animals were observed from a safe distance to ensure that oviposition proceeded normally.

The collection of samples was conducted under Maio Biodiversity Foundation guidelines and by the permits of the Environmental National Authority DNA (Direção Nacional do Ambiente). Research protocols were performed per the IUCN Policy Statement on Research Involving Species at Risk of Extinction [41] approved by the 27th Meeting of IUCN Council, Gland Switzerland, 14 June 1989, and the Sea Turtle Research Techniques Manual [42].

### 4.3. Isolation and Identification of Gram-Negative Bacteria

After pre-enrichment in Buffered Peptone Water (Oxoid, Basingstoke, UK) at 37 °C for 24 h, aerobic and facultative anaerobic Gram-negative bacteria were isolated from collected samples using Glutamate Starch Red Phenol (GSP) Agar plates supplemented with 100,000 UI penicillin g/L (Merck, Darmstadt, Germany) and MacConkey Agar (Oxoid, Basingstoke, UK), incubated at 37 °C for 24–48 h [2]. Positive bacterial colonies were isolated in Columbia agar supplemented with 5% sheep blood (bioMérieux, Marcy-l’Étoile, France). Isolates were characterised regarding their macro and microscopic morphology, Gram staining, catalase test and oxidase reaction. For further characterisation, 19 Gram-negative bacilli were selected and identified through the biochemical identification galleries API 20E and 20NE (bioMérieux, Marcy-l’Étoile, France), according to the manufacturer’s instructions.

### 4.4. Evaluation of Isolates’ Antimicrobial Resistance Profile

Isolates’ susceptibility profiles regarding 12 different antimicrobials commonly and globally used in veterinary and human medicine belonging to distinct classes were determined using the disk diffusion method according to the Clinical and Laboratory Standards Institute (CLSI) guidelines [43]. The tested antibiotics (Oxoid, Basingstoke, Hamp, UK, and Mast Group, Bootle, UK) were: amikacin (AK, 30 µg), cefoperazone (CFP, 75 µg), ceftazidime (CAZ, 30 µg), ciprofloxacin (CIP, 5 µg), enrofloxacin (ENR, 5 µg), gentamicin (GM, 120 µg), imipenem (IMP, 10 µg), meropenem (MEM, 10 µg), ofloxacin (OFX, 5 µg), piperacillin (PIP, 100 µg), tetracycline (T, 30 µg), and tobramycin (TOB, 10 µg), as described elsewhere [2,44]. The reference strains *Escherichia coli* ATCC^®^ 25922^TM^ and *Pseudomonas aeruginosa* ATCC^®^ 27853 ^TM^ were used as quality control. The inhibition zones were measured, and isolates were scored as susceptible, intermediate, and resistant, according to the CLSI guidelines [43]. Intrinsic resistances were taken into consideration, according to Magiorakos et al. [15]. A 10% replica was performed in independent days, by repeating the antimicrobial susceptibility testing of 10% randomly selected isolates [43].

Multiple antimicrobial resistance (MAR) indices were calculated for the selected isolates as follows: nº. antimicrobials to which isolates were resistant/nº. antimicrobials tested. A MAR index equal or greater than 0.20 was used as a cut-off to differentiate between high and low-risk contamination [29].

### 4.5. Evaluation of Isolates’ Virulence Profile

Isolates were characterised regarding their phenotypic virulence profile by assessing the production of enzymes associated with bacterial pathogenic potential.

Hemolysins production was evaluated using Columbia Agar with 5% sheep blood [30].

DNase activity was assessed using DNase Agar supplemented with 0.005% methyl green (VWR, Leuven, Belgium), using *Aeromonas hydrophila* ATCC^®^ 7966^TM^ and *Escherichia coli* ATCC^®^ 25922^TM^ as positive and negative controls, respectively [45].

Lecithinase activity was determined using Tryptic Soy Agar (VWR, Leuven, Belgium), supplemented with 10% egg yolk emulsion (VWR, Leuven, Belgium). Positive and negative controls, *Pseudomonas aeruginosa* ATCC^®^ 27853^TM^ and *Escherichia coli* ATCC^®^ 25922^TM^ were used, respectively [46].

Gelatinase activity was detected using Nutrient Gelatin Agar (Oxoid, Basingstoke, UK), using *Pseudomonas aeruginosa* ATCC^®^ 27853^TM^ and *Escherichia coli* ATCC^®^ 25922^TM^ as positive and negative controls, respectively [30].

Biofilm production ability was assessed resorting to Congo Red Agar plates, composed of Brain Heart Infusion broth (VWR, Leuven, Belgium), Bacteriological Agar (VWR, Leuven, Belgium), and 0.0008% Congo Red indicator (Sigma-Aldrich, St. Louis, USA). *Enterococcus faecium* ATCC^®^ 35667^TM^ and *Escherichia coli* ATCC^®^ 25922^TM^ were, respectively, used as positive and negative controls [47,48].

Protease activity was analysed, resorting to Skim Milk Agar (Oxoid, Basingstoke, UK), using *Pseudomonas aeruginosa* ATCC^®^ 27853^TM^ and *Staphylococcus aureus* ATCC^®^ 29213^TM^ as positive and negative controls, respectively [49].

Lipase activity was tested using Spirit Blue Agar (Difco, Detroit, USA) supplemented with 0.25% Tween^®^ 80 (AppliChem GmbII, Darmstadt, Germany) and 25% olive oil (commercial), using *Pseudomonas aeruginosa* ATCC^®^ 27853^TM^ and *Staphylococcus aureus* ATCC^®^ 29213^TM^ as positive and negative controls, respectively [30]. For testing all virulence factors, the plates were incubated at 25 °C for 24 h.

The virulence indices were calculated for the tested isolates, as follows: nº. positive virulence factors/nº. virulence factors tested [33]. A virulence index equal to or higher than 0.50 was used as a cut-off to evaluate the threat levels for the selected isolates, regarding their pathogenic potential [33]. According to Singh et al. [33], isolates were categorised as a high threat-isolates with virulence and MAR indices greater than or equal to the cut-off values (MAR index ≥ 0.20, V. Index ≥ 0.50); moderate threat-isolates having virulence index ≥ 0.50 but MAR index < 0.20; and no threat-isolates having virulence and MAR indices below the cut-off values.

## Figures and Tables

**Table 1:** Antimicrobial resistance of bacterial isolates from oral, cloacal, and egg content swab samples of 33 loggerhead turtles.

Antimicrobial Class	Antimicrobial Compound (Dose)	Number of Isolates Tested		
		Susceptible	Intermediate	Resistant
	Amikacin (30 µg)	19	0	0
Aminoglycosides	Gentamicin (120 µg)	19	0	0
	Tobramycin (10 µg)	19	0	0
Carbapenems	Meropenem (10 µg)	18	1	0
	Imipenem (10 µg)	13	6	0
Cephalosporins	Cefoperazone (75 µg)	17	1	1
	Ceftazidime (30 µg)	18	1	0
Fluoroquinolones	Ciprofloxacin (5 µg)	18	1	0
	Enrofloxacin (5 µg)	16	2	1
	Ofloxacin (5 µg)	19	0	0
Tetracyclines	Tetracycline (30 µg)	17	0	2
Ureidopenicillins	Piperacillin (100 µg)	18	0	1

**Table 2:** Selected isolates’ antimicrobial resistance profile.

Isolate Number	Animal ID (Flipper Tag)	Sample Type	Isolate Identification	Resistance Profile		MAR Index
				Intermediate	Resistant	
1	276/030	C	*A. hydrophila/caviae*	IMP; ENR	CFP	0.33
2	786/785	C	*B. vesicularis*	IMP	-	0.08
3	329/328	E	*B. cepacia*	ENR; CFP	-	0.17
4	276/030	C	*S. putrefaciens*	-	-	0.00
5	049/050	C	*S. putrefaciens*	-	T	0.08
6	045/046	E	*S. putrefaciens*	-	-	0.00
7	045/046	C	*S. putrefaciens*	IMP	-	0.08
8	072/073	C	*S. putrefaciens*	IMP	-	0.08
9	276/030	E	*V. alginolyticus*	-	-	0.00
10	276/030	E	*V. alginolyticus*	-	-	0.00
11	060/061	C	*V. alginolyticus*	-	-	0.00
12	503/504	O	*V. alginolyticus*	-	T	0.08
13	330/331	C	*E. cloacae*	-	-	0.00
14	049/050	C	*E. cloacae*	CIP	ENR; PIP	0.25
15	045(046	C	*M. morganii*	MEM	-	0.08
16	045/046	E	*M. morganii*	-	-	0.00
17	276/030	O	*M. morganii*	IMP	-	0.08
18	503/504	C	*M. morganii*	IMP	-	0.08
19	228/229	C	*Citrobacter* spp.	CAZ	-	0.08

Cloaca (C), oral cavity (O), egg content (E), imipenem (IMP), enrofloxacin (ENR), cefoperazone (CFP), tetracycline (T), ciprofloxacin (CIP), piperacillin (PIP), meropenem (MEM), ceftazidime (CAZ), multiple antimicrobial resistance index (MAR Index).

**Table 3:** Selected isolates’ virulence profile.

Isolate Number	Isolate Identification	Virulence Profile							V. Index
		HEM	DNase	LIP	LEC	PT	GEL	BF	
1	*A. hydrophila/caviae*	β	+	+	+	+	−	24	0.86
2	*B. vesicularis*	α	+	+	−	+	+	24	0.86
3	*B. cepacia*	α	+	+	−	+	+	−	0.43
4	*S. putrefaciens*	α	+	+	+	+	−	24	0.86
5	*S. putrefaciens*	α	+	+	+	−	+	-	0.43
6	*S. putrefaciens*	β	+	+	+	+	−	24	1.00
7	*S. putrefaciens*	α	+	+	Inc	+	−	24	0.86
8	*S. putrefaciens*	α	+	+	Inc	+	−	24	0.86
9	*V. alginolyticus*	α	+	−	Inc	+	−	48	0.57
10	*V. alginolyticus*	α	+	−	−	+	−	24	0.57
11	*V. alginolyticus*	α	+	−	−	−	−	24	0.43
12	*V. alginolyticus*	α	+	−	−	−	−	24	0.43
13	*E. cloacae*	α	+	+	−	−	−	48	0.57
14	*E. cloacae*	α	+	+	−	+	−	48	0.71
15	*M. morganii*	β	+	+	−	−	−	−	0.43
16	*M. morganii*	α	+	+	−	−	−	−	0.43
17	*M. morganii*	α	+	+	−	−	−	−	0.43
18	*M. morganii*	α	−	+	−	−	−	24	0.57
19	*Citrobacter* spp.	α	−	+	−	−	−	72	0.43

Alpha-haemolysis (α), beta-haemolysis (β), positive (+), negative (−), inconclusive (Inc), haemolysin (HEM), lipase (LIP), lecithinase (LEC), protease (PT), gelatinase (GEL), biofilm (BF).

## Data Availability

Data are contained within the article or Supplementary Materials.

## Supplementary Materials

The following are available online at https://www.mdpi.com/article/10.3390/antibiotics10070771/s1, Table S1: Sampling data.
