# Supplementary material for: Antibiotic Resistance and Virulence Profiles of Gram-Negative Bacteria Isolated from Loggerhead Sea Turtles (Caretta caretta) of the Island of Maio, Cape Verde
Source: Antibiotics (Basel). 2021 Jun 24;10(7):771. doi: 10.3390/antibiotics10070771 (PMC8300689; doi:10.3390/antibiotics10070771)
Supplement: Supplementary file 1 [file antibiotics-10-00771-s001.zip › antibiotics-1252400-supplementary.pdf]

Supplementary Material - 1

Table S1. Sampling data.

Sample nº.	Date	Time	Beach/ local	Animal flipper tag ID	PIT	Sample type
1	8/1/2019	21h51	"Praiona", "Pedro Vaz"	326/327	9.81098(...)258	cloaca
2	8/1/2019	21h51	"Praiona", "Pedro Vaz"	326/327	9.81098(...)258	oral cavity
3	8/1/2019	21h51	"Praiona", "Pedro Vaz"	326/327	9.81098(...)258	egg
4	8/1/2019	23h00	"Praiona", "Pedro Vaz"	329/328	-	cloaca
5	8/1/2019	23h00	"Praiona", "Pedro Vaz"	329/328	-	oral cavity
6	8/1/2019	23h00	"Praiona", "Pedro Vaz"	329/328	-	egg
7	8/1/2019	23h21	"Praiona", "Pedro Vaz"	330/331	-	cloaca
8	8/1/2019	23h21	"Praiona", "Pedro Vaz"	330/331	-	oral cavity
9	8/1/2019	23h21	"Praiona", "Pedro Vaz"	330/331	-	egg
10	8/1/2019	6h10	"Praiona", "Pedro Vaz"	335/334	9.81098(..)250	cloaca
11	8/1/2019	6h10	"Praiona", "Pedro Vaz"	335/334	9.81098(...)250	oral cavity
12	8/1/2019	6h10	"Praiona", "Pedro Vaz"	335/334	9.81098(...)250	egg
13	8/2/2019	22h30	“Areia Preta”, “Praia Gonçalo”	104/098	9.81098(...)598	cloaca
14	8/2/2019	22h30	“Areia Preta”, “Praia Gonçalo”	104/098	9.81098(...)598	oral cavity
15	8/2/2019	22h30	“Areia Preta”, “Praia Gonçalo”	104/098	9.81098(...)598	egg
16	8/2/2019	23h32	“Cozinha fácil”, “Praia Gonçalo”	024/025	9.81098(...)803	cloaca
17	8/2/2019	23h32	“Cozinha fácil”, “Praia Gonçalo”	QMI 024/025	9.81098(...)803	oral cavity
18	8/2/2019	23h32	“Cozinha fácil”, “Praia Gonçalo”	QMI 024/025	9.81098(...)803	egg
19	8/2/2019	23h55	“Cozinha fácil”, “Praia Gonçalo”	QMI 064/065	9.81098(...)690	cloaca
20	8/2/2019	23h55	“Cozinha fácil”, “Praia Gonçalo”	QMI 064/065	9.81098(...)690	oral cavity
21	8/2/2019	23h55	“Cozinha fácil”, “Praia Gonçalo”	QMI 064/065	9.81098(...)690	egg
22	8/2/2019	01h26	“Areia Preta”, “Praia Gonçalo”	QMI 099/100	9.81098(...)752	cloaca
23	8/2/2019	01h26	“Areia Preta”, “Praia Gonçalo”	QMI 099/100	9.81098(...)752	oral cavity
24	8/2/2019	01h26	“Areia Preta”, “Praia Gonçalo”	QMI 099/100	9.81098(...)752	egg
25	8/2/2019	02h10	“Areia Preta”, “Praia Gonçalo”	QMI 784/783	9.81098(...)606	cloaca
26	8/2/2019	02h10	“Areia Preta”, “Praia Gonçalo”	QMI 784/783	9.81098(...)606	oral cavity
27	8/2/2019	02h10	“Areia Preta”, “Praia Gonçalo”	QMI 784/783	9.81098(...)606	egg
28	8/2/2019	02h40	“Areia Preta”, “Praia Gonçalo”	QMI 786/785	9.81098(...)330	cloaca
29	8/2/2019	02h40	“Areia Preta”, “Praia Gonçalo”	QMI 786/785	9.81098(...)330	oral cavity
30	8/2/2019	02h40	“Areia Preta”, “Praia Gonçalo”	QMI 786/785	9.81098(...)330	egg

Passive Integrated Transponder (PIT).

Table S1. *cont.*

Sample nº.	Date	Time	Beach/ local	Animal flipper tag ID	PIT	Sample type
31	8/4/2019	21h50	“Areia Preta”, “Praia Gonçalo”	QMI 039/038	-	cloaca
32	8/4/2019	21h50	“Areia Preta”, “Praia Gonçalo”	QMI 039/038	-	oral cavity
33	8/4/2019	21h50	“Areia Preta”, “Praia Gonçalo”	QMI 039/038	-	egg
34	8/5/2019	00h34	“Areia Preta”, “Praia Gonçalo”	QMI 051/052	9.81098(...)652	cloaca
35	8/5/2019	00h34	“Areia Preta”, “Praia Gonçalo”	QMI 051/052	9.81098(...)652	oral cavity
36	8/5/2019	00h34	“Areia Preta”, “Praia Gonçalo”	QMI 051/052	9.81098(...)652	egg
37	8/5/2019	01h20	“Areia Preta”, “Praia Gonçalo”	QMI 028/029	9.81098(...)018	cloaca
38	8/5/2019	01h20	“Areia Preta”, “Praia Gonçalo”	QMI 028/029	9.81098(...)018	oral cavity
39	8/5/2019	01h20	“Areia Preta”, “Praia Gonçalo”	QMI 028/029	9.81098(...)018	egg
40	8/6/2019	22h13	“Cozinha fácil”, “Praia Gonçalo”	QMI 062/063	-	cloaca
41	8/6/2019	22h13	“Cozinha fácil”, “Praia Gonçalo”	QMI 062/063	-	oral cavity
42	8/6/2019	22h13	“Cozinha fácil”, “Praia Gonçalo”	QMI 062/063	-	egg
43	8/6/2019	22h30	“Cozinha fácil”, “Praia Gonçalo”	QMI 060/061	-	cloaca
44	8/6/2019	22h30	“Cozinha fácil”, “Praia Gonçalo”	QMI 060/061	-	oral cavity
45	8/6/2019	22h30	“Cozinha fácil”, “Praia Gonçalo”	QMI 060/061	-	egg
46	8/6/2019	22h47	“Cozinha fácil”, “Praia Gonçalo”	QMI 064/065	-	cloaca
47	8/6/2019	22h47	“Cozinha fácil”, “Praia Gonçalo”	QMI 064/065	-	oral cavity
48	8/6/2019	22h47	“Cozinha fácil”, “Praia Gonçalo”	QMI 064/065	-	egg
49	8/6/2019	23h20	“Cozinha fácil”, “Praia Gonçalo”	QMI 066/067	-	cloaca
50	8/6/2019	23h20	“Cozinha fácil”, “Praia Gonçalo”	QMI 066/067	-	oral cavity
51	8/6/2019	23h20	“Cozinha fácil”, “Praia Gonçalo”	QMI 066/067	-	egg
52	8/6/2019	00h00	“Cozinha fácil”, “Praia Gonçalo”	QMK 067/068	-	cloaca
53	8/6/2019	00h00	“Cozinha fácil”, “Praia Gonçalo”	QMK 067/068	-	oral cavity
54	8/6/2019	00h00	“Cozinha fácil”, “Praia Gonçalo”	QMK 067/068	-	egg
55	8/6/2019	01h40	“Cozinha fácil”, “Praia Gonçalo”	KMJ 504/503	-	cloaca
56	8/6/2019	01h40	“Cozinha fácil”, “Praia Gonçalo”	KMJ 504/503	-	oral cavity
57	8/6/2019	01h40	“Cozinha fácil”, “Praia Gonçalo”	KMJ 504/503	-	egg
58	8/8/2019	21h53	“Areia Preta”, “Praia Gonçalo”	QMI 073/072	9.81098(...)147	cloaca
59	8/8/2019	21h53	“Areia Preta”, “Praia Gonçalo”	QMI 073/072	9.81098(...)147	oral cavity
60	8/8/2019	21h53	“Areia Preta”, “Praia Gonçalo”	QMI 073/072	9.81098(...)147	egg

Passive Integrated Transponder (PIT).

Table S1. *cont.*

Sample nº.	Date	Time	Beach/ local	Animal flipper tag ID	PIT	Sample type
61	8/8/2019	22h30	"Areia Preta", "Praia Gonçalo"	770/771	9.81098(...)885	cloaca
62	8/8/2019	22h30	"Areia Preta", "Praia Gonçalo"	770/771	9.81098(...)885	oral cavity
63	8/8/2019	22h30	"Areia Preta", "Praia Gonçalo"	770/771	9.81098(...)885	egg
64	8/8/2019	23h16	"Areia Preta", "Praia Gonçalo"	773/772	9.81098(...)591	cloaca
65	8/8/2019	23h16	"Areia Preta", "Praia Gonçalo"	773/772	9.81098(...)591	oral cavity
66	8/8/2019	23h16	"Areia Preta", "Praia Gonçalo"	773/772	9.81098(...)591	egg
67	8/8/2019	01h40	"Areia Preta", "Praia Gonçalo"	229/228	-	cloaca
68	8/8/2019	01h40	"Areia Preta", "Praia Gonçalo"	229/228	-	oral cavity
69	8/8/2019	01h40	"Areia Preta", "Praia Gonçalo"	229/228	-	egg
70	8/8/2019	02h10	"Areia Preta", "Praia Gonçalo"	050/258	-	cloaca
71	8/8/2019	02h10	"Areia Preta", "Praia Gonçalo"	050/258	-	oral cavity
72	8/8/2019	02h10	"Areia Preta", "Praia Gonçalo"	050/258	-	egg
73	13/8/19	01h04	"Cozinha fácil", "Praia Gonçalo"	094/095	-	cloaca
74	13/8/19	01h04	"Cozinha fácil", "Praia Gonçalo"	094/095	-	oral cavity
75	13/8/19	01h04	"Cozinha fácil", "Praia Gonçalo"	094/095	-	egg
76	13/8/19	02h38	"Cozinha fácil", "Praia Gonçalo"	049/050	-	cloaca
77	13/8/19	02h38	"Cozinha fácil", "Praia Gonçalo"	049/050	-	oral cavity
78	13/8/19	02h38	"Cozinha fácil", "Praia Gonçalo"	049/050	-	egg
79	13/8/19	03h10	"Cozinha fácil", "Praia Gonçalo"	046/045	-	cloaca
80	13/8/19	03h10	"Cozinha fácil", "Praia Gonçalo"	046/045	-	oral cavity
81	13/8/19	03h10	"Cozinha fácil", "Praia Gonçalo"	046/045	-	egg
82	13/8/19	03h20	"Cozinha fácil", "Praia Gonçalo"	276/035	-	cloaca
83	13/8/19	03h20	"Cozinha fácil", "Praia Gonçalo"	276/035	-	oral cavity
84	13/8/19	03h20	"Cozinha fácil", "Praia Gonçalo"	276/035	-	egg
85	13/8/19	03h44	"Cozinha fácil", "Praia Gonçalo"	277/278	-	cloaca
86	13/8/19	03h44	"Cozinha fácil", "Praia Gonçalo"	277/278	-	oral cavity
87	13/8/19	03h44	"Cozinha fácil", "Praia Gonçalo"	277/278	-	egg
88	16/8/19	22h43	"Cozinha fácil", "Praia Gonçalo"	298/505	-	cloaca
89	16/8/19	22h43	"Cozinha fácil", "Praia Gonçalo"	298/505	-	oral cavity
90	16/8/19	22h43	"Cozinha fácil", "Praia Gonçalo"	298/505	-	egg

Passive Integrated Transponder (PIT).

Table S1. *cont.*

Sample nº.	Date	Time	Beach/ local	Animal flipper tag ID	PIT	Sample type
91	16/8/19	00h36	"Cozinha fácil", "Praia Gonçalo"	284/283	-	Cloaca
92	16/8/19	00h36	"Cozinha fácil", "Praia Gonçalo"	284/283	-	Oral cavity
93	16/8/19	00h36	"Cozinha fácil", "Praia Gonçalo"	284/283	-	Egg
94	16/8/19	01h36	"Cozinha fácil", "Praia Gonçalo"	285/286	-	Cloaca
95	16/8/19	01h36	"Cozinha fácil", "Praia Gonçalo"	285/286	-	Oral cavity
96	16/8/19	01h36	"Cozinha fácil", "Praia Gonçalo"	285/286	-	Egg
97	16/8/19	02h37	"Areia preta", "Praia Gonçalo"	288/287	-	Cloaca
98	16/8/19	02h37	"Areia preta", "Praia Gonçalo"	288/287	-	Oral cavity
99	16/8/19	02h37	"Areia preta", "Praia Gonçalo"	288/287	-	Egg

Passive Integrated Transponder (PIT).
